# “About sixty per cent I want to do it”: Health researchers’ attitudes to, and experiences of, patient and public involvement (PPI)—A qualitative interview study

**DOI:** 10.1111/hex.12883

**Published:** 2019-03-29

**Authors:** Anne‐Marie Boylan, Louise Locock, Richard Thomson, Sophie Staniszewska

**Affiliations:** ^1^ Nuffield Department of Primary Care Health Sciences University of Oxford Oxford UK; ^2^ Health Services Research Unit University of Aberdeen Aberdeen UK; ^3^ Institute of Health and Society Newcastle University Newcastle‐upon‐Tyne UK; ^4^ Warwick Research in Nursing Warwick Medical School University of Warwick Coventry UK

**Keywords:** health and medical research, patient and public involvement, qualitative interviews

## Abstract

**Background:**

Funders, policy‐makers and research organizations increasingly expect health researchers in the UK to involve patients and members of the public in research. It has been stated that it makes research “more effective, more credible and often more cost efficient.” However, the evidence base for this assertion is evolving and can be limited. There has been little research into how health researchers feel about involving people, how they go about it, how they manage formal policy rhetoric, and what happens in practice.

**Objective:**

To explore researchers’ experiences and perceptions of patient and public involvement (PPI).

**Methods:**

Semi‐structured interview study of 36 health researchers (both clinical and non‐clinical), with data collection and thematic analysis informed by the theoretical domains framework.

**Results:**

In the course of our analysis, we developed four themes that encapsulate the participants’ experiences and perceptions of PPI. Participants expressed ambivalence, cynicism and enthusiasm about PPI, an activity that creates emotional labour, which is both rewarding and burdensome and requires practical and social support. It is operationalized in an academic context influenced by power and incentives.

**Discussion and conclusions:**

Researchers’ experiences and attitudes towards patient and public involvement are a key factor in the successful embedding of involvement within the wider research culture. We call for a culture change that supports the development of effective organizational approach to support involvement.

## 1 BACKGROUND

Patient and Public Involvement (PPI) in research is increasingly expected by funders, policy‐makers and research organizations. This is an international movement; in some countries, such as Canada and the USA, the term engagement is preferred to involvement, but the intention is the same, namely to do research “with” people rather than “to” or “for” them.

The UK's Chief Medical Officer has asserted that PPI can make “research more effective, more credible and often more cost efficient.”^1^ One systematic review^2^ found evidence of increased recruitment and retention, as well as some evidence that patient involvement helped in securing funding, in designing study protocols and in selecting relevant outcomes. A systematic review specifically on recruitment and retention found that PPI interventions had a modest but significant effect.^3^ In primary care, Blackburn et al^4^ found most reported impact was on the design of studies and writing participant information, with few reported impacts on gaining funding or on the management and conduct of research.

However, the practice of PPI and the evidence base is still emergent, and the quality of impact reporting is inconsistent.^5^ Considerable debate remains as to what can and should be measured, and how we define PPI as an “intervention.”^6^, ^7^ Fundamental differences in understanding of the goals and value of PPI result in very different approaches to understanding impact—is it a technical process to improve the effectiveness, acceptability and feasibility of medical research, or is it rather about democratic rights and empowerment, or even an essential challenge to what constitutes “evidence”?^8^, ^9^, ^10^ Qualitative research can provide insights into how PPI is implemented (or not) and how it may affect research,^11^, ^12^ but it cannot give us a definitive account of impact. Staley, et al^13^ have argued that we may be better considering impact on researchers—over time, on their behaviours, emotions and ways of thinking—than on specific pieces of research. Thus, health and medical researchers are exhorted to implement PPI as a way to improve the quality and effectiveness of their work, based on a, as yet, somewhat limited evidence base. This may be felt to be in tension with a predominant emphasis on evidence‐based medicine, and the expectation that interventions should be rigorously evaluated before widespread implementation. Researchers’ beliefs and behaviours are of course also fundamental to whether, how and at what stages of the research cycle PPI takes place. The cycle of tokenism presents a risk: researchers who are unconvinced about the benefits involve people in a half‐hearted or superficial way, reinforcing their belief that it makes little difference.^14^

There have been few studies of how researchers have responded to the pressure to move towards a more participatory model of knowledge production.^15^ The privileging of scientific expertise over lay experience has been remarkably resilient, and it has been noted that researchers are reluctant to cede control over research.^16^, ^17^ Despite some interest in, and support for, PPI in theory, researchers may feel apprehensive and reluctant to change their practice, given other professional and institutional priorities. 18 Boaz et al's^15^ interview study with 19 biomedical research centre scientists found little evidence of any change in these attitudes and concludes that “science remains the preserve of scientists, with patients and the public invited to ‘tinker at the edges’.” (p.592).

A focus group and interview study of the perspectives of 24 Australian researchers^19^ is one of a small number of studies to focus explicitly on researchers’ experiences of PPI. The report identifies a mix of practical barriers to involvement (such funding time and contributor involvement, lack of skills and difficulty finding people) as well as more relational issues, including anxiety and defensiveness; fear of antagonistic or “difficult” patients; and power hierarchies both within academia, and between academics and patients.

Some of these factors have been previously reported in other studies of the process and impact of PPI, several of which have included interviews or surveys with researchers. Wilson et al's^20^ realist evaluation across 22 nationally funded research projects in the UK, for example, finds that there are continuing uncertainties for researchers in terms of the purpose and value of PPI, how and at what stages to involve people, and whether/how to assess impact. Further contested areas include whether people can have a place in basic science research and whether people become too research‐aware to be considered “authentically lay.” They conclude that whilst there are good examples of well‐embedded involvement in research, there are also “tensions that must be navigated in balancing moral and methodological imperatives” (p.98).

However, much previous research has focused primarily on implementation of, and barriers to, PPI rather than explicitly investigating how health researchers feel about this expectation to involve people; how they go about it; their emotions, fears and hopes; and their personal perspectives on managing potential dissonance between formal policy rhetoric, the evidence and what happens in practice. This study aimed to fill that gap.

## 2 METHODS

We conducted a qualitative interview study, approved by Berkshire Research Ethics Committee [Ref 12/SC/0495]. The study was guided by an expert advisory panel which included patients and family carers with experience of PPI, PPI coordinators and representatives from the National Institute for Health Research's (NIHR) advisory group on PPI, INVOLVE. The study was funded by the Oxford NIHR Biomedical Research Centre through its PPI cross‐cutting theme.

Thirty‐six participants were recruited through a wide range of avenues from across England, Scotland and Wales. We aimed for a maximum variation sample,^21^ seeking variation across types of research (clinical, medical scientists and social scientists); research design (eg researchers using trials, cohort studies, qualitative methods); levels of seniority (from early career researcher to principle investigator); degree of experience of PPI; and age, gender and ethnicity. We also included some people working as research managers or involvement coordinators. Table 1.

**Table 1:** Sample characteristics

Characteristic	Number of participants
Gender	
Female	22
Male	13
Ethnicity	
White British	24
White European	3
White Other	6
British Asian	1
Age	
26‐44 y	14
45‐64 y	18
Unspecified	3
Role	
Clinical and medical scientific researchers (CMSR)	14
Social scientists and health services researchers (SS/HSR)	18
PPI coordinators	3
Experience of involving people in research	
<1 y	1
1‐5 y	10
6‐10 y	8
>10 y	13
Unspecified	3

Participants were interviewed at a location of their choice, usually their academic workplace. Interviews were video‐ or audio‐recorded, depending on consent, and transcribed verbatim.

## 3 THEORETICAL LENS

The implementation of PPI requires researchers to change their existing behaviours and practices and, in the case of new researchers, adapt to this relatively new practice in a culture that is also adapting. There are numerous frameworks for seeking to understand behaviour change and organizational implementation. We adopted Michie et al's^22^ theoretical domains framework (TDF) to guide both data collection and thematic analysis. Its focus on professional behaviour, evidence‐based medicine and the adoption of new practices seemed particularly relevant to this study. We drew on, but adapted, the suggested questions to develop prompts for our own interview guide. During analysis, we also adopted a micro‐meso‐macro level lens, incorporating wider factors into our developing understanding of implementing PPI, that is we looked at individual, organizational and broader cultural factors (eg funders).

Interviews were semi‐structured. Participants were initially invited to talk about their current role and how they had first started involving patients and the public in their research. Prompts included both invitations to give further detail about how and why they had involved people, and prompts derived from the theoretical domains framework. In practice, some prompts were dropped or merged (Box 1).

BOX 1 **Using the TDF as a basis for an interview guide**

1

**Table:** 

Each of the domains as originally published was accompanied by a set of detailed underlying constructs, and suggested interview questions, which we developed into an interview guide. We revised this after early interviews revealed it was difficult to ask the questions in this way. For example, we merged questions on evidence into one.	
TDF questions	Revised for interview guide
Knowledge: “what do they think the evidence is?” Social/professional role: “what do they think about the credibility of the source [of a guideline]?” Beliefs about consequences: “does the evidence suggest that doing x is a good thing?”	Tell me a bit about your views of the evidence base for PPI
Social Influences: “To what extent do social influences facilitate or hinder x?” “Will they observe others doing x?”	How do your colleagues feel about PPI? Do they approve of it or disagree?

The data were analysed using an iterative thematic approach,^23^ supported by data management software (NVivo). Data were coded and categorized using a framework that was developed deductively based on the TDF, but we also included inductive codes that arose from the interviews. Further inductive analysis of the TDF coding was conducted to ensure that we looked beyond it and into the views of the participants. This further analysis was used to generate themes that encapsulate participants’ experiences. In Table 2 below, we present the themes and demonstrate what domains of the TDF were used in generating them.

**Table 2:** TDF to theme development

Theme	TDF domains
Practical and social support	Skills; Memory, attention and decision processes; Motivation and goals; Behavioural regulation; Environmental context and resources
Rewards and burdens of emotional labour	Skills; Motivation and goals, Emotions; Social influences; Behavioural regulation
Ambivalence, cynicism and enthusiasm	Knowledge; Memory, attention and decision processes; Motivation and goals; Beliefs about consequences
Academia, power and incentives.	Social/professional role and identity; Beliefs about capabilities; Beliefs about consequences; Memory, attention and decision processes; Social influences; Environmental context and resources; Nature of the behaviours

## 4 FINDINGS

Table 2 presents an overview of the themes and the domains of the TDF that were used to construct them.

### 4.1 Practical and social support

Patient and Public Involvement required significant administrative labour. Participants talked about the lack of practical support to do this work, and the time and effort diverted from other activities.

Embedding PPI in practice was described as a complex process. It required a range of resources, including time, money, space to interact with contributors, skill development and refinement, and an infrastructure to support PPI (including PPI coordinators). Generally, no additional researcher time was allocated for the work associated with involvement. This raised questions about its value and importance.

I don’t get any extra allowance to do PPI… it's just something extra that I'm having to fit on top of everything else. Kathleen, 29, Senior researcher, SS/HSR

Some participants said they needed help to manage the additional work it added to already stretched schedules.

I'd love admin support just so that we had everything streamlined… not me searching around for the right tailored information that they would need and typing it up myself. So that sort of thing would be really, really good but I will drop things deliberately to deal with PPI because it is incredibly important. Maria, Senior lecturer and researcher, age withheld, CMSR

Participants talked about the value of PPI coordinators, staff whose role was to support contributors and facilitate the process of PPI:

There’s a particular skill in being able to span those two worlds, the academic research world and the lay world and to act as some kind of translator between the two and I think there is something there that’s a particular skill…Robert, 49, senior researcher, SS/HSR

On a macro level, wider cultural mechanisms were seen as significant drivers for PPI. Participants discussed the impact of organizations that drive PPI, including funders, like the NIHR and its national advisory group on PPI, INVOLVE. On a meso level, organizational culture and having role models or experienced colleagues were seen as important in supporting involvement:

…There's a cultural support for involving various patients and members of the public in the organisation and I think that’s very helpful to be part of. And even kettle conversations whilst making a cup of tea and you're sharing challenges and experiences with people who understand that, are a great resource so I do think the culture of the organisation is an important support for being able to do this properly… It would be much more difficult if you were on your own doing this without a supportive community of people. Dan, 48, Senior researcher, SS/HSR

Involving contributors not only required funding and time, but also social support from colleagues and opportunities to learn from each other, as this new and largely unfamiliar way of working emerged. Senior “buy‐in” was also seen as important as a means to legitimize the practice of PPI; when senior staff held negative views about the importance of PPI, it complicated successful involvement.

…Don’t underestimate how tricky it can be when a more senior person has … more set views on what PPI can and can't do… If someone in a more senior position isn’t willing to open their mind and be receptive to genuine change, isn’t willing to accept differences to what they want to do, then there's kind of no point in you trying really because I think everyone needs to be working to the same goal for it be effective. Lily, 26, Associate research fellow, SS/HSR

### 4.2 Rewards and burdens of emotional labour

In addition to the time costs outlined previously, participants discussed both the costs and rewards of another aspect of involvement, emotional labour. Emotional labour required participants to manage their feelings and emotional responses in line with their professional context. It was undertaken by researchers, both in terms of supporting PPI contributors and in dealing with their own personal emotions evoked by involvement.

Participants often talked with enthusiasm about the rewards of PPI. Jennifer said “…It raises my enthusiasm to battle the challenges of getting research funding.” Others described how it enriched their experience of work making it more fun and moving in addition to helping them produce more interesting and worthwhile research.

This emotional labour and its associated costs were seen as necessary, important but also rewarding. Participants described how it had a positive impact on them emotionally as well as on their work:

It makes you feel good about what you do… The more engagement I’ve had with members of the public the more A) the more fun it is but also more useful I feel the stuff we do is. Mike, 59, Professor, CMSR

Challenges faced by researchers included suppressing their opinions, having to be polite when they felt they wanted to be otherwise and wrestling with the need to involve people in tasks that researchers had spent a significant amount of time training to do. This was particularly true for qualitative researchers, who felt there was a perception that contributors could be more readily involved in qualitative research. Discussions on these points were often conflicted. The researchers who discussed them held positive views about the value and worth of PPI, but raised these points as challenges to implementing it alongside research work.

Mm about sixty percent I want to do it. The bits of me that don’t want to do it are the kind of, 'Oh god I've got to be polite to people when I’m not in the mood to be’ [laughs] – all that kind of stuff. Denise, 47, Senior research officer, SS/HSR

The researchers described how PPI can increase their level of responsibility at work, including adding a level of emotional responsibility that may not be prevalent in all other aspects of academia, and an added responsibility to take care of people which is generally not part of a professional academic role. Taking care of people included public contributors. For principal investigators, this also meant taking care of more junior staff who, through involvement, could become exposed to emotionally upsetting life‐experiences.

Bringing emotion to the table was seen as part of the PPI role: “emotion is the power that they bring to the situation” (Amanda). In the world of objectivity and science, patients, the public and carers were seen as humanizing research that may otherwise be purely academic: “[it's] my work – to someone else it's their everyday, it's really emotional, a big issue for them.” (Lily).

Emotionally supporting PPI contributors can be burdensome and is magnified when contributors are unwell (physically or mentally). Abi (50, Research Fellow in PPI, SS/HSR) described the impact of this:

…It ceases to be an academic exercise, you’re working with real people…and something seemingly innocuous can just trigger something for somebody… You have responsibility as such to take care of the people you’re working with. And I think that’s a very personal emotional cost because these aren’t other researchers; these are patients and members of the public.

Researchers were also subject to criticism by PPI contributors, and some described feeling upset and insulted:

[There is] this idea that we need PPI because actually we're all these kind of robotic, unfeeling people who don’t understand what patients go through….I've spoken to hundreds of patients; I spend all my time…exploring the impact on them, and you're telling me that I don’t know anything about it…..It's almost a bit of a professional insult and a bit of a personal insult. Margaret, 32, Research Fellow, SS/HSR

Abi described the pressure to “deliver, but there's also the managing and holding of their expectations and their emotional responses to the process, and dealing with things when things go wrong. Dealing with things when they go right…ultimately you are going to elicit strong emotions in people.” She went on to describe PPI as “a real rollercoaster.”

### 4.3 Ambivalence, cynicism and enthusiasm

Participants identified a wide range of skills necessary for involvement, including relational skills, communication skills (accessible language, listening, translating academic concepts), open‐mindedness and empathy, in addition to administrative and organizational skills. These were often described as “soft” skills. Kelly said PPI is “all about building relationships…. You need to be able to build relationships; you need an enormous amount of enthusiasm for it.” Social and interpersonal skills were seen as necessary for involvement, but this could lead to the delegation of PPI to those who are seen to have such skills (see also “academia, power and incentives”), meaning that the additional associated work and responsibility for PPI was unevenly distributed.

Participants held a range of views and feelings about PPI. They also cited their colleagues’ views when discussing what it was like to involve contributors. These ranged from cynical or sceptical to ambivalent to positive. Rose (32, Senior Researcher, SS/HSR) explained the tension she felt about PPI contributors performing tasks that required a skilled researcher:

I've got to be really careful as to what I say and do…PPI's really trendy at the minute… Patients should be researchers – why don’t we just [effing] bring a load of patients to come and sit round my desk? Why did I bother doing a PhD? Do you know what I mean? So it's like really difficult because these people are quite capable people, but they’ve not had the training, they’ve not worked as a researcher.

Colleagues’ views, and general organizational culture, were seen to be significant in determining both the practice and the impact of involvement. For example, Michelle (39, Clinical Researcher, CMSR) described how she did not share her involvement activities with colleagues as they were extremely sceptical about it:

Quite honestly, the very senior people think this is a waste of time and a box‐ticking exercise. And a lot of what I've been doing I’ve been keeping it quiet, because I don’t want anyone to tell me that I'm wasting my time.

The participants were motivated to involve contributors for a range of reasons. In terms of professional motivation, PPI was seen useful for endorsing research ideas, reducing waste by ensuring research is focused on patient priorities and that research designs were appropriate and acceptable to potential participants. For instance, Julie (29, Clinical Scientist, CMSR) described how a study was not recruiting sufficient numbers of participants. After having it reviewed by a PPI group, the research team made a number of changes that improved it.

…Coming in from an academic starting point where you’re … not necessarily used to writing for a lay audience, you tend to err on the side of complexity rather than keeping things simple and to the point. And I realised in hindsight there was a lot of unnecessary description and overly complex words.

Researchers often felt ill‐equipped in terms of resources and training for PPI. This increased stress for some, but as Kathleen explained this might be offset by low expectations from colleagues:

It's a tiny bit nerve‐wracking to be leading something that you don’t have expertise in. So in that way, yeah, I do feel the stress of being responsible for something that I don’t feel that expert at all in……There's no pressure to do an amazing job on it because people aren’t expecting a lot from it…

Despite the labour associated with undertaking PPI, the participants also discussed the rewards it brought. For those who discussed this, it was often described as a positive part of their work, something that improved their research and made them feel good about doing it. They often described the experience of PPI positively, using terms like “energising.” There was an emotional return for involvement; it can provide positive feedback in an otherwise slow process of research.

As a researcher we have a long timescale before we get the sort of buzz of the paper being published. Even longer timescale before we get the actual impact, and we'll probably never know the people on whom we've made an impact. At least by being involved with PPI groups throughout the course, we get a bit of that positive feedback as to what the benefit might be and for me that’s very, it raises my enthusiasm to battle the challenges of getting research funding. Jennifer, 46, Research Professor, CMSR

### 4.4 Academia, power and incentives

The “publish or perish” culture of academia was highlighted frequently in researchers’ narratives, particularly in establishing the importance of PPI. Some questioned where PPI sits in relation to core measures of academic success, including publishing, obtaining funding and the Research Excellence Framework, which assesses the quality and impact of research in the UK. However, they acknowledged that PPI could enhance research by generating research questions, improving study design and recruitment, and diversifying views in analysis, for instance, resulting in it being seen as good for career development.

Academics face multiple competing priorities, and these were seen to be differently experienced according to career stage. In particular, early career researchers were seen as having to engage with all the traditional tasks of academia, plus becoming adept at involving contributors (often without appropriate resources). Given the cultural importance of the “publish or perish” culture, there was a question of incentives to do PPI over other competing priorities,.

…All academia is interested in is the Research Excellence Framework and the publication you get at the end of it. So the whole PPI and the added value and the changes that introduces, and the resources …you have to put into that, I'm not sure that that is at all valued in the current sort of academic tick‐boxing. Although you know clearly the university certainly wants to see outreach but that’s not quite the same perhaps, or not viewed in quite the same way. Jennifer, 46, Research Professor, CMSR

Being responsible for PPI meant researchers felt diverted from career‐enhancing activities, from which other colleagues were benefiting.

I definitely would want PPI to be involved in all my studies but I don’t want to be the sole person responsible for every time because that’s going to take away from research time and then I'll be doing the PPI for my colleagues and they'll be able to do more research and get more publications out of it… Kathleen, 29 Senior Researcher, SS/HSR

Fixed‐term, short‐term contracts compound the problems associated with involvement. Typically, more junior staff are responsible for PPI and they are likely to be employed on such contracts. This can have a negative effect on PPI as Rachel (31, Research Fellow, SS/HSR) explained, “…if you're on a fixed‐term contract of twelve months or six months it can be quite hard to build a relationship that's meaningful.” This had a consequential impact for mid‐career or senior staff, who then had to maintain the established PPI. This was particularly salient where formalized groups had been formed (eg patient panels).

If you’ve got a research assistant who’s employed for a project who can do that for you, that’s great, but the project ends …So then you're left doing it yourself and it is really hard to keep it going because it's on top of whatever else you're doing. And it's not funded. Grace, 51, Associate Professor, CMSR

As Eddie (36, Research Fellow, SS/HSR) explained, those responsible for PPI appear to be those who lack power “at the bottom of the chain within that academic pyramid.” This heightens the potential for a disconnect between PPI and the research, possibly limiting the impact contributors can have.

In addition to the association between PPI and being junior and on fixed‐term contracts, participants observed a gender divide in terms of who bears the administrative and emotional labour of PPI. Not only was there an observation that women were usually responsible for PPI, but the participants suggested that this might be due to a wider perception about the skills needed and the value of PPI.

I find it interesting that so much of the researchers who are involved are female. You go to [academic] meetings about public involvement and you get one man and 20 women and is that because…. they’re softer skills about communication and listening and empathy? Gill, age/job role withheld, SS/HSR

Gill went on to say that in another corner of her department there is a male researcher who champions PPI and questioned whether she could then draw conclusions from her previous observation. However, championing PPI is not the same as doing the labour of PPI: several other participants talked about senior men championing PPI, but not doing the work of involvement.

This kind of thinking had potentially negative consequences for the success of involvement, which involved a lot of complex intervention and skilled facilitation on the part of the researcher.

I wonder sometimes if that’s why they give it to kind of the younger researchers because it's like, “Oh basically you're a nice young girl aren’t you, you're inoffensive and you're very nice to people so you can go and do the PPI,” as if that means you can sit here and run what is essentially a collaboration event, that you can deal with what might be strong personalities or conflicting personalities… Margaret, 32 Research Fellow, SS/HSR

Some participants also observed that those responsible for PPI were often qualitative researchers. This was particularly true for mixed methods research or in clinical trials and possibly because the associated skills are similar to those needed for PPI. However, the lack of understanding of the differences between qualitative research and PPI was frustrating for the qualitative researchers we interviewed.

…It’s much more difficult for people to get involved in quantitative research for lots and lots of reasons… Whereas qualitative work in health services research is much more accessible so for them it’s easy to get involved with. Maybe the sorts of people who come into qualitative research have those sorts of empathetic outlooks anyway. Gill, age/job role withheld, SS/HSR

## 5 DISCUSSION

This study explored the implementation of PPI from the perspective of health and medical researchers demonstrating that it is a highly complex undertaking that can be both beneficial and burdensome. Its implementation is affected by a range of micro, meso and macro level factors; it is operationalized within a “publish or perish” culture that can result in it being deprioritized, causing tension for those who see its benefit. Attitudes towards PPI range from cynical to ambivalent to positive. It is often the responsibility of women and junior staff, and is particularly complicated when it lacks support from senior colleagues.

These findings are particularly relevant in light of Staley's^13^ call for research on the impact of PPI to focus on the impacts on researchers. In contrast to previous research,^15^, ^18^ we found that researchers were willing to change their practice, but that this was complicated by the culture in which they operate and a myriad of associated factors, including notions of power, academic structures and career development. This context can limit the successful implementation of guidance produced by organizations promoting PPI.

In debates about PPI in research, the dominant discourse is one in which researchers hold the power and contributors do not, or at least have to find ways to negotiate power for themselves in an unequal relationship.^24^ The problem with this discourse is that it overlooks how hierarchical academic life can be, and how power may be unevenly distributed amongst researchers; between research disciplines; and between researchers and the organizations and processes which shape their lives, such as university employers, funding bodies, ethics committees and peer review. It also overlooks the possibility that researchers may feel that they have considerably less power vis‐à‐vis patients/public than is sometimes assumed. Equally, more junior researchers may feel that they do not have the power needed to ensure involvement activities are embedded in research, particularly if they are working with a more senior person who treats it as a tick‐box exercise.

Our findings also indicate that responsibility for PPI is not equitably distributed, with female and junior researchers often being tasked with it. There was a sense that junior staff starting their careers were more open to the notion of PPI and felt it was part of the culture of academia. Drawing on the concept of “emotional labour,” the fact that women appear to have more responsibility for it than men may be unsurprising. In 1983, Arlie Hochschild^25^ coined the term to describe the work of managing emotions that was required by some professions, predominantly undertaken by women. It has previously been considered in higher education in the USA,^26^, ^27^ but not with regard to PPI, which arguably extends the emotional reach of academia. As our findings indicate, PPI involves a significant amount of emotional labour from taking care of contributors, to suppressing emotions, to operating in a context that deprioritizes involvement. Devolving responsibility to women may be linked to socially constructed notions of what it is to be a woman and the perception that they are more likely to be skilled in these tasks.

At a more structural level, however good the relationship with individual patients and members of the public may be, researchers may resent the implication that their expertise is devalued. This may be more threatening to professional identity in some fields than in others, reinforcing inter‐disciplinary hierarchies. There was an erroneous perception reported here that patient involvement in qualitative interviewing and analysis is uncontroversial, whereas it is not generally assumed that patients’ lived experience qualifies them to get involved in statistical analysis. The fact that such disciplinary hierarchies may also map (albeit imperfectly) onto gender compounds the challenge to professional identity.

Research leaders who acted as positive role models and created an environment where PPI was commonplace helped reduce dissonance between “doing PPI” and “being a good academic.” In other contexts, researchers who chose to pursue PPI very actively might feel they were going against the tide and damaging their careers. Others might feel that their career was being damaged for them by senior researchers giving signals that PPI was an unimportant task, and by extension, the people in the organization to whom they delegated it were also unimportant. Our findings suggest that this view has not been entirely overcome.

Pressure from funding bodies, and especially a strong steer from the NIHR, has undoubtedly fostered a climate in which PPI is seen as a requirement rather than an option. However, it is questionable whether this should be understood as truly “game‐changing,” or rather a stimulus to “gaming,” in which ticking boxes are literally as well as metaphorically required. The truth, as ever, lies probably somewhere in between, whereby researchers are genuinely making efforts to do more and better PPI, but at the same time finding it irksome and not always expecting to live up to the promises made in grant application forms in order to get the funding.

There were clear examples in our data of people holding very critical views of PPI but not feeling empowered to voice these publicly. In reality, PPI may be seen by researchers and funders alike as unlikely to be a deciding factor in funding decisions, in contrast with scientific criteria. This in turn further affects how researchers are seen within their organization if they are perceived to be spending too much time on PPI.

### 5.1 Strengths and limitations

This is one of the first papers to draw on the TDF and implementation literature to look at PPI in health and medical research. As such, it considers researchers’ views on *if* or *why* they should involve patients and public, and on *how* they do it and the range of factors affecting adoption. The TDF is a comprehensive framework for understanding meso and micro factors in implementation. However, it excludes the macro level, which is problematic for the implementation of PPI as wider socio‐political factors are key in this debate. Nonetheless, we used a micro‐meso‐macro approach to analysis to ensure we explored these crucial factors.

The TDF proved to be a useful lens in exploring the implementation of PPI. In analysis, it provided helpful direction and we did not consider it unduly limiting, as we were guided by it rather than following it deductively. On a practical note, we found it difficult to implement as an interview guide and had to modify it, as detailed in Box 1.

The design of this study allowed participants to freely express their views on involvement, leading to new findings about researchers’ experiences of implementing PPI. Our sample was limited to participants working in England, Scotland and Wales and, although we achieved a good range in terms of career stage and length of experience of PPI, we were unable to recruit equal numbers of female and male staff. In spite of these limitations, key findings from this work may be transferrable to other settings.

### 5.2 Implications

From our analysis, it is clear that more support is needed for PPI—practical support, including funding for PPI input to develop bids (which is not always currently available); time, including longer deadlines for funding calls and more dedicated assistance (eg administrative support) with conducting PPI activities. Intragroup and intergroup support in the form of researchers sharing practice within and across organizations is also vital. We call for a cultural change to challenge the tick‐box approach that may have resulted from making PPI an operational requirement. Much like the Athena Swan initiative that is attempting to improve academic culture for women in science, an initiative to embed PPI could reinforce its value and promote it to a more prominent and important role. Some evidence of attempts to challenge research culture has been made in recent years, including publications focused on how to involve contributors in clinical trials^28^ and qualitative research,^29^ and in co‐producing their involvement.^30^ However, what is needed is a dedicated initiative aimed at solidifying involvement as part of research culture. Such an initiative could draw on existing training and should include shared learning offered by academics and PPI contributors; sharing practice and experiences is a valuable form of training that might make PPI more accessible. Such a scheme may have a positive effect on culturally embedding PPI.

## CONFLICTS OF INTEREST

The authors have no conflict of interests to declare.
